# Self-Regulating Wind Speed Adaptive Mode Switching for Efficient Wind Energy Harvesting Towards Self-Powered Wireless Sensing

**DOI:** 10.3390/mi17030373

**Published:** 2026-03-19

**Authors:** Ruifeng Li, Chenming Wang, Yiao Pan, Jianhua Zeng, Youchao Qi, Ping Zhang

**Affiliations:** 1 School of Mechanical and Electrical Engineering, Guilin University of Electronic Technology, No. 1 Jinji Road, Guilin 541004, China; liruifeng8107@163.com (R.L.); wangchenmingg@163.com (C.W.); 15014373635@163.com (Y.P.); 2 CAS—Center for Excellence in Nanoscience, Beijing Key Laboratory of Micro-Nano Energy and Sensor, Beijing Institute of Nanoenergy and Nanosystems, Chinese Academy of Sciences, Beijing 101400, China; 3 School of Fashion and Textiles, The Hong Kong Polytechnic University, Hong Kong SAR, China

**Keywords:** self-regulating mechanism, triboelectric nanogenerator, wind energy harvesting, power management circuit, wireless temperature-humidity sensing

## Abstract

Wind energy harvesting based on triboelectric nanogenerators (TENGs) is a promising solution for powering distributed Internet of Things (IoT) nodes, yet its practical efficiency and stability are often hindered by the fluctuating and unpredictable nature of wind. Here, we propose a self-regulating TENG (SR-TENG) that leverages the synergistic effects of centrifugal, elastic, and frictional forces to automatically switch between non-contact and contact modes based on wind speed. This configuration achieves an ultra-low start-up wind speed of 0.86 m/s, ensures sustainable high-performance output across a broad wind speed range, and exhibits excellent durability with no observable performance degradation during 23,000 s of continuous operation at 375 rpm. Systematic structural optimization enables the SR-TENG to reach a peak open-circuit voltage of 140 V, a short-circuit current of 12.5 μA, and a transferred charge of 300 nC at 375 rpm. When integrated with a customized power management circuit, the system delivers a 30.39-fold increase in effective output power at a 1 MΩ load and a 4-fold faster charging rate for a 10 μF capacitor. For practical validation, the harvested ambient wind energy successfully powers a wireless temperature-humidity sensor for real-time cloud data transmission. These results highlight that the SR-TENG holds great potential for advanced wind energy harvesting and self-powered sensing applications in distributed IoT systems.

## 1. Introduction

With the rapid development of the Internet of Things (IoT) and distributed sensing nodes, global energy demand consumption will continue to increase [1,2]. However, traditional power supply methods based on wired connections and batteries are limited by issues such as finite lifespan, high maintenance costs, and significant environmental burdens, making it difficult to meet sustainable demands [3,4,5,6,7]. Harvesting energy from the surrounding environment has become a key way to promote the widespread application of the IoT [8,9,10]. Environmental energy primarily includes solar energy [6,11,12], vibration energy [13,14,15], human motion energy [16,17,18], and wind energy [19,20,21,22,23]. Specifically, wind energy is a very common mechanical energy in nature. Due to its advantages such as abundant reserves, all-weather operational capability, and broad environmental adaptability, it is one of the most promising energy sources in environmental energy collection [24,25,26]. Therefore, the efficient wind energy conversion technology is of crucial importance for establishing sustainable and distributed clean energy supply systems.

The traditional method of converting wind energy into electricity is based on the principle of electromagnetic induction and turbine structures [27,28]. However, this method has problems such as large device volume and mass, high installation cost, difficulty in start-up under low wind speed, which severely limit their applicability in regions with weak winds and unstable wind conditions [29,30,31], making it difficult to adapt to miniaturized or large-scale distributed scenarios. In contrast, the triboelectric nanogenerator (TENG) converts mechanical energy into electricity through the coupled effect of triboelectrification and electrostatic induction, emerging as a highly promising micro-energy harvesting technology [31,32]. This is because the TENG has inherent advantages under low-frequency and irregular mechanical excitation, such as lightweight structure, strong material adaptability, and low environmental dependence [33,34,35,36,37,38]. This device has proven highly effective in scavenging ambient energy. However, existing wind energy TENGs generally have problems such as high start-up wind speed, unstable output under fluctuating wind speeds, and a lack of adaptive mode adjustment, which severely limit their wide application in real-world outdoor environments. Liu et al. [39] achieved stable intermittent wind energy conversion through magnetic force control, generating continuous and regular electrical power. Yong et al. [40] proposed a multi-stage strategy with dual-axis automatic switching, enabling efficient wind energy capture across a wide wind speed range of 2.2–16 m/s. Zou et al. [41] introduced a robust triboelectric nanogenerator (TENG-SS) based on a self-regulating strategy, reducing the starting wind speed to 2.4 m/s and enabling self-powered wind speed sensing with adjustable strategies. Chen et al. [42] designed an ultra-stable rotational TENG with a built-in traction rope structure, which utilizes centrifugal force, increasing with rotational speed, to autonomously transition between contact and non-contact modes. Fu et al. [43] proposed a water-driven ultra-stable rotational TENG that can automatically switch modes, operating in contact mode at low speeds and non-contact mode at high speeds, while also achieving charge excitation. However, existing wind energy TENGs generally face issues such as suboptimal starting wind speeds, unstable output under fluctuating wind speeds, and a lack of adaptive regulation mechanisms that balance sensitivity and durability during high- and low-speed transitions, which severely limit their widespread application in real-world outdoor environments.

Herein, a self-regulating triboelectric nanogenerator (SR-TENG) is designed to automatically toggle between non-contact and contact modes based on wind speed variations. This smart mechanism effectively eliminates friction at low speeds, enabling an ultra-low start-up threshold of 0.86 m/s, while utilizing centrifugal force to engage intimate contact at higher speeds for maximized electrical output. Through systematic structural optimization, the device achieves a peak open-circuit voltage of 140 V, a short-circuit current of 12.5 μA, and a transferred charge of 300 nC at 375 rpm. To further boost practical utility, a power management circuit is integrated, which amplifies the effective output power over 30-fold (at 1 MΩ) and quadruples the charging speed for a 10 μF capacitor. The integrated system successfully drives wireless temperature-humidity sensors via Bluetooth for real-time data transmission. These results highlight the SR-TENG’s immense potential as a sustainable power solution for self-powered IoT nodes, distributed environmental monitoring, and smart transportation systems.

## 2. Materials and Methods

### 2.1. Fabrication of the SR-TENG

The overall dimensions of the SR-TENG are 260 mm in diameter and 160 mm in height. The key components of the SR-TENG include the wind cup, rotor, stator, cover plate, and bearings (POM608, Rongtu, Liaocheng, China). The wind cup is made of aluminum alloy. The stator, cover plate, rotor, and slider were all 3D-printed using polylactic acid (PLA) material with a 3D printer (SLA, HI-800, Zhongke, Jinan, China). The slider was attached with eight pieces of polytetrafluoroethylene (PTFE) film (length: 80 mm, width: 15 mm, thickness: 0.08 mm) as the friction layer. Sixteen pieces of copper foil (length: 43 mm, width: 20 mm, thickness: 0.1 mm) were alternately fixed on the inner surface of the stator as electrode layers. The cover plate and stator, as well as the stator and rotor, are connected by two bearings, ensuring more thorough contact between them.

### 2.2. Characterization and Measurement

The performance testing platform of the SR-TENG consists of an NI data acquisition card, a programmable electrometer (Keithley 6514, Keithley, Solon, OH, USA), a computer, and a stepper motor (5IK40RGN-C, PC MOTOR, Suzhou, China). The stepper motor (5IK40RGN-C) simulates wind energy and drives the SR-TENG to rotate. The output signal of the SR-TENG is collected through the programmable electrometer (Keithley 6514) and the NI data acquisition card, with data processing performed using LabVIEW (KEITHLEY 6514 Nanogenerator Measurement Software Ver2.02). In the application of wireless real-time temperature and humidity monitoring, a commercial hair dryer (RC-7136, RIWA, Ningbo, China) was used to provide the required excitation wind, and the wind speed was measured and evaluated using a commercial anemometer (Fluke 923, Fluke, Everett, WA, USA). A microcircuit chip (LTC3588, Zave, Shantou, China) serves as the energy management module. The temperature and humidity sensor module (GY-BLE39, GY, Guilin, China) was used to measure external environmental parameters and wirelessly transmit data via Bluetooth.

## 3. Results and Discussion

### 3.1. Structural Design and Operational Principle of the SR-TENG

**Figure 1:**
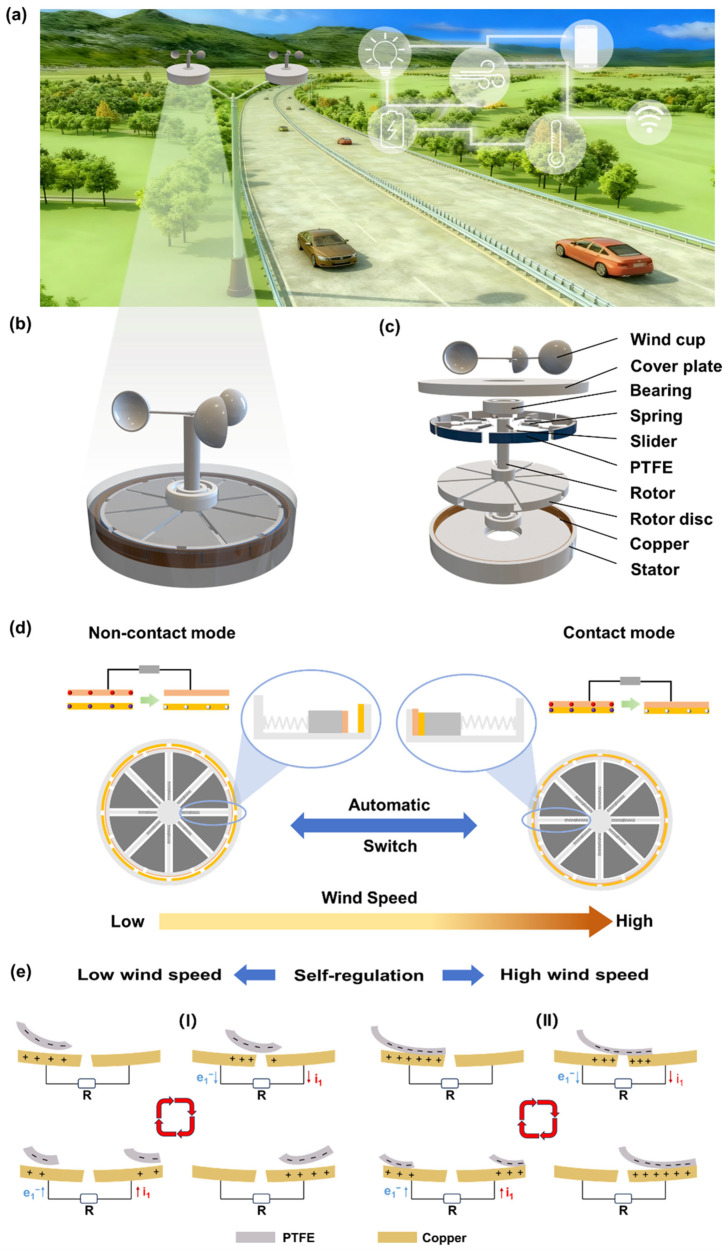
The structure and working principle of SR-TENG. (**a**) Schematic diagram of the self-powered intelligent transportation application scenario in mountainous areas utilizing high-speed wind energy. (**b**) Three-dimensional schematic illustration of the SR-TENG. (**c**) Exploded-view schematic diagram illustrating the assembly of the SR-TENG. (**d**) Working mechanism of SR-TENG self-regulation. The red dots represent the negative charges accumulated on the PTFE membrane surface after contact electrification. The purple dots represent the positive charges left behind after contact electrification. The white dots represent the negative charges induced by the electric field after separation. The gray box denotes the load component along the current path, which is used to consume or measure the generated electric energy. The green arrows indicate the dynamic direction of charge separation. (**e**) Schematic diagram illustrating the working principle of the SR-TENG. The red arrows represent the direction of current flow. The blue arrows represent the direction of electron flow. The circular arrows indicate one working cycle of the TENG.

Mountainous areas are known to contain abundant wind energy resources, and the SR-TENG is capable of capturing this energy and converting it into electricity. As illustrated in Figure 1a, the SR-TENG installed on streetlights in mountainous regions can harvest wind energy and store it in energy storage units, thereby powering small electronic devices such as traffic signal lights, warning lights, temperature and humidity sensors, and other small electronic devices. This setup creates a sustainable traffic assistance system that integrates distributed micro-energy supply and autonomous environmental sensing.

Figure 1b,c and Figure S1 illustrate the structural model, three-dimensional schematic diagram, and photograph of the SR-TENG. As a rotary TENG, it primarily comprises three coaxially mounted components: a wind cup, a rotor, and a stator. Specifically, the wind cup is fixed to the central shaft to drive the rotor. The rotor assembly consists of a disc, a central shaft, springs, and sliders. Each slider is connected to the shaft via a spring and features a 0.08 mm-thick polytetrafluoroethylene (PTFE) film bonded to its outer surface as the friction layer. Positioned beneath the rotor, the stator holds 16 uniformly distributed copper electrodes (0.1 mm thick), with adjacent electrodes connected in a staggered-parallel configuration.

During operation, the centrifugal force generated by rotation is transmitted to the sliders. Through the interplay of centrifugal force, friction, and the spring’s restoring force, the sliders extend outward, enabling adaptive regulation of the contact-separation state between the PTFE layer and the copper electrodes. To ensure stability and coaxial alignment, the rotor is mounted between the cover plate and the stator using nested nylon bearings, which effectively minimizes rotational resistance and enhances reliability. This structural design allows the SR-TENG to achieve self-regulation in response to varying wind speeds driven by rotation-induced centrifugal force.

The SR-TENG can automatically adjust its operating mode in response to varying wind speeds (Figure 1d). When the wind is low, the rotor rotates at a relatively low velocity, and the generated centrifugal force is insufficient to overcome the spring’s restoring force and frictional resistance, thus keeping the slider in a retracted state with a small air gap maintained between the copper electrode and the PTFE film. Under this condition, the SR-TENG operates in the non-contact mode, where no direct physical contact is established between the electrode and the film. At high speed, the rotor speed elevates accordingly, leading to a gradual rise in centrifugal force that exceeds the spring’s restoring force and frictional resistance. Consequently, the slider is pushed outward, bringing the copper electrode into contact with the PTFE film and thereby switching the device into the contact mode. By virtue of this wind speed-adaptive switching mechanism, the SR-TENG can automatically adjust its working mode in accordance with wind speed fluctuations, achieving low-energy startup at low wind speeds and maximizing electrical output at high wind speeds, which ultimately optimizes the efficiency of wind energy harvesting.

Figure 1e illustrates the working principle of the SR-TENG under different wind speeds. At low wind speeds, the device operates in the non-contact mode, with a tiny gap maintained between the copper electrodes and the PTFE film (Figure 1e(I)). Even without physical touching, due to the significant difference in electron affinity between the copper electrode and PTFE, the latter exhibits a superior electron-attracting ability. Consequently, a stable layer of negative charges is maintained on the PTFE surface, establishing an electrostatic field. As the rotor spins, this field drives electrons back and forth between adjacent electrodes through the external circuit via electrostatic induction. This process generates a continuous, albeit smaller, pulsed alternating current (AC), effectively harvesting energy from gentle breezes that would otherwise be wasted. When the wind speed increases, the stronger centrifugal force pushes the sliders outward, forcing the copper electrodes to press directly against the PTFE film. This shifts the device into contact mode (Figure 1e(II)). The intense physical friction between the layers triggers the triboelectric effect, which pumps much more charge onto the surfaces. Now, with a significantly higher surface charge density, the combined action of contact electrification and electrostatic induction works together. As the electrodes slide over the film, they drive a much stronger and faster flow of electrons, producing a significantly enhanced AC output compared to the non-contact stage, thereby significantly improving the electrical output performance under strong wind conditions.

### 3.2. Optimization of Output Performance of SR-TENG

**Figure 2:**
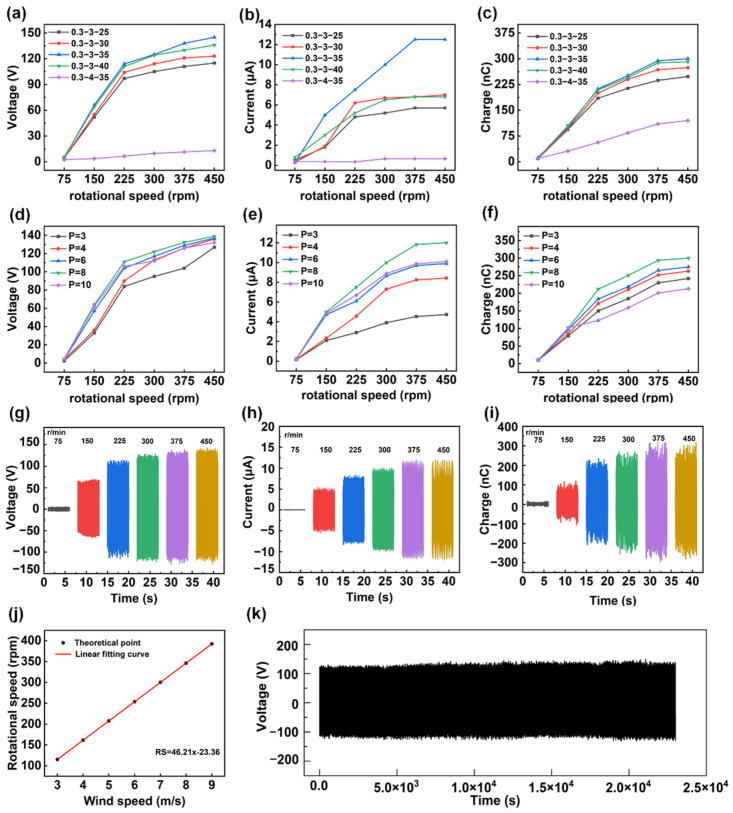
Experimental optimization of the output performance of the SR-TENG: (**a**–**c**) Eff–ct of spring outer diameter and length on SR-TENG output. (**d**–**f**) Eff–ct of the number of electrode pairs on SR-TENG output. (**g**–**i**) Eff–ct of different rotational speeds on SR-TENG output. (**j**) Corresponding curve of wind speed and rotational speed. (**k**) Open-circuit voltage during continuous operation for 23,000 s.

In order to optimize the output performance of the SR-TENG, we systematically investigated the effects of key physical parameters—including spring specifications, the number of electrode pairs, and rotational speed on its output characteristics through a series of controlled (Figure 2a–c and Figure S2). With the wire diameter fixed at 0.3 mm, different combinations of outer diameter and length were compared (outer diameter of 3 mm with lengths of 25–40 mm, and outer diameter of 4 mm with a length of 35 mm). According to Note S1, when the outer diameter is 3 mm, increasing the spring length *n* from 25 mm to 35 mm moderately reduces the spring stiffness k, resulting in larger radial displacement of the slider at the same rotational speed. This enhances the effective contact between the friction layers, leading to a significant improvement in output performance. However, when the length is further increased to 40 mm, the output exhibits a clear saturation trend, and even the current shows limitations. This is because the excessively reduced stiffness, although able to maintain the charge quantity, weakens the axial restoring force of the spring, causing a delayed retraction of the slider and a decrease in the effective contact–separation frequency. Meanwhile, increasing the outer diameter from 3 mm to 4 mm, the stiffness b comes more sensitive to the change in outer diameter, especially at high rotational speeds. The softer 4 mm spring with lower stiffness is unable to provide sufficient restoring force to counteract the rapidly increasing centrifugal force, leading to excessive deformation. Such deformation not only causes instability in the slider’s motion trajectory but may also induce vibrations due to insufficient stiffness, resulting in uneven load distribution at the contact interface and ultimately reducing the charge transfer efficiency at the friction surface, causing a noticeable decrease in output. Overall, the 0.3-3-35 spring delivered the best performance across all tested rotational speeds. Taking 450 rpm as an example, the output voltage, current, and transferred charge reached approximately 140 V, 12 μA, and 300 nC, respectively. This behavior can be attributed to a balanced interplay among centrifugal force, the spring restoring force, and the frictional resistance of the slider. Considering a single slider, the centrifugal force during rotation can be expressed as:

(1) $$F_{c}=mr\omega^{2}$$

m is the equivalent mass of the slider and the attached friction layer, r is the effective radius of the slider relative to the rotation center, and ω is the angular velocity. The restoring force produced by the spring can be written as:

(2) $$F_{s}=kx$$

where k is the spring stiffness, and x is the axial deformation of the spring. The frictional force between the slider and the guiding slot is denoted as:

(3) $$F_{f}=\mu N$$

where μ is the coefficient of friction, and N is the normal load. The mechanical equilibrium condition in the radial direction can be written as:

(4) $$F_{c}=F_{s}+F_{f}$$

At a relatively low rotational speed ω, where $F_{c}<F_{s}+F_{f}$, the slider remains stationary. A certain gap is maintained between the PTFE friction layer and the copper electrode layer, allowing the SR-TENG to operate in a non-contact mode with low frictional resistance, which facilitates easy start-up. As the rotational speed increases, the rise in ω leads to a rapid increase in $F_{c}$. When $F_{c}$ exceeds the sum of the spring restoring force and frictional resistance, the slider is pushed outward, and the PTFE friction layer is pressed against the copper electrode, transitioning the SR-TENG from non-contact to contact mode, as shown in Figure S3. At this stage, the effective contact area between PTFE and the copper electrode increases significantly [44,45], leading to more efficient interfacial charge separation and electrostatic induction processes. Consequently, the output voltage, current, and transferred charge are all markedly enhanced. If the spring stiffness is too high or the length is too short, a higher rotational speed is required to achieve the equilibrium condition of Equation (4). As a result, the device remains in the non-contact state for an extended period within the operating wind speed range, limiting output. Conversely, when the spring is too soft or too long, although SR-TENG easily enters contact mode, excessive deformation and contact instability occur at high rotational speeds, resulting in an incomplete contact-separation process, which also suppresses output. Therefore, balancing both the start-up performance and contact stability, the 0.3-3-35 type spring was selected for subsequent experiments. On this basis, the effect of the number of electrode pairs *P* on the output performance of SR-TENG was further studied (Figure 2d–f and Figure S4). When the number of electrode pairs was increased from 3 to 8, the output performance improved overall, with the open-circuit voltage remaining stable at approximately 140 V, while the short-circuit current increased from about 4.7 μA to 12.2 μA, and the corresponding transferred charge rose from about 250 nC to 300 nC. The effective area of the electrode can be approximated as:

(5) $$S=PS_{0}$$

where $S_{0}$ is the effective area corresponding to a single electrode. The open-circuit voltage for SR-TENG is expressed as:

(6) $$V_{oc}=\frac{△\sigma_{sc}\cdot S}{C}$$

where $△\sigma_{sc}$ represents the equivalent charge density transferred to the electrodes during each contact-separation cycle, and C denotes the equivalent capacitance between the electrodes. The short-circuit charge and transferred charge satisfy:

(7) $$I_{sc}\approx △Q\cdot f,\;△Q=△\sigma_{sc}S$$

where f is the contact-separation frequency. From Equations (5)–(7), it can be seen that, with other parameters unchanged, increasing *P* will enhance the effective area S, thereby increasing △Q and $I_{sc}$, which is consistent with the experimental trend observed in Figure 2d–f from *P* = 3 to *P* = 8. However, when the number of electrode pairs increases to 10, the output slightly decreases. This drop is likely because the electrodes are packed too closely together. The high density creates a very strong electric field in the tiny gaps between adjacent electrodes. This intense field can break down the air, causing sparks or invisible leakage paths that allow the accumulated charges to escape, rather than flowing through the circuit to generate power.

**Figure 3:**
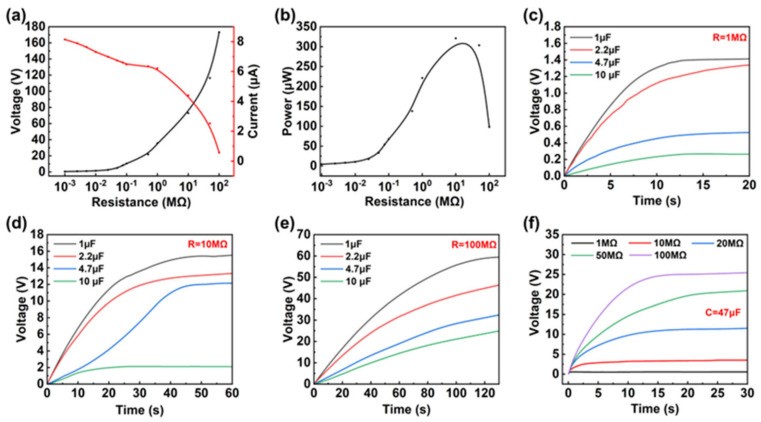
Load characteristics of the SR-TENG: (**a**) Output voltage and current of the SR-TENG u-der different load resistances. (**b**) Output power of the SR-TENG as a function of load resistance. (**c**–**e**) Inf–uence of an identical load resistance on the output voltage across different storage capacitors. (**f**) Influence of different load resistances on the output voltage for an identical storage capacitor.

After optimizing the spring (0.3-3-35) and the number of electrode pairs (*P* = 8), the output characteristics of SR-TENG at different rotational speeds were tested (Figure 2g–i). When the rotational speed was increased from 75 rpm to 150 rpm, the voltage, current, and transferred charge all exhibited a sudden increase, corresponding to the transition of the device from non-contact mode to contact mode. To quantify this transition process, the theoretical critical threshold rotational speed was calculated to be 130 rpm according to Equation (4) and Note S2. It was measured that, when the motor speed increased directly from 75 rpm to 150 rpm (Figure S5a), the output voltage showed a significant rise at approximately 2.3 s, indicating that the SR-TENG transitioned from non-contact mode to contact mode, and the voltage waveform continued to increase without any voltage drop. Although the experimental set rotational speed of 150 rpm is slightly higher than the theoretical threshold, the stability of the waveform confirms that the centrifugal force always overcomes the spring restoring force and frictional resistance, preventing the system from returning to the non-contact mode. However, when the motor speed decreased from 150 rpm to 75 rpm (Figure S5b), the voltage drop slowed significantly, and the duration of the drop increased, indicating that the transition from contact mode back to non-contact mode occurred at a different speed compared to the transition from non-contact mode to contact mode. Nonetheless, this does not affect the output performance of the SR-TENG. When the rotational speed was further increased from 375 rpm to 450 rpm, the output voltage, current, and charge all tended to saturate, indicating that stable and full contact between the PTFE and copper electrodes had been achieved. Ultimately, at a rotational speed of 375 rpm, a maximum open-circuit voltage of approximately 140 V, a maximum short-circuit current of approximately 12.5 μA, and a transferred charge of approximately 300 nC were achieved. Based on the above parametric studies, we selected the structural parameters (spring 0.3-3-35, *P* = 8, rotation speed 375 rpm) to achieve the optimal output performance. Figure S6 shows the open-circuit voltage and short-circuit current of the SR-TENG at different wind speeds, with the maximum open-circuit voltage reaching 131 V and the short-circuit current being 11.2 μA. To further verify the relationship between rotation speed and wind speed, when the rotation speed is higher than 150 rpm, and the wind speed exceeds 3 m/s, both the short-circuit current and the rotation speed exhibit an approximately linear dependence (Figure S7), thereby establishing the correlation between rotation speed and wind speed (Figure 2j). A wind speed of approximately 8.6 m/s is required to achieve a rotational speed of 375 rpm. At this speed, after 23,000 s of continuous operation (Figure 2k), the output voltage maintained exceptionally high stability throughout the entire testing period, with no observable performance degradation. Furthermore, the performance of previously reported TENGs was analyzed and compared with that of the SR-TENG, as summarized in Table S1 [29,43,46,47,48,49]. The data demonstrate that the SR-TENG exhibits certain advantages in terms of durability. The optimal matched load of the SR-TENG was determined by connecting external resistors with different values to the circuit. The output voltage was measured in parallel, whereas the output current was measured in series. Figure 3a shows the outcomes of tests conducted to measure voltage and current under varying loads. At 375 rpm, increasing load resistance leads to an increase in open-circuit voltage, while short-circuit current rapidly decreases due to the high internal resistance characteristics of the TENG. Consequently, the output power initially increases and then decreases. As shown in Figure 3b and Figure S8, the device achieves an instantaneous maximum power output of 320 μW under a 10 MΩ matched load in contact mode. In non-contact mode, the maximum power output is approximately 20 μW under a 500 MΩ matched load. Additionally, the power conversion efficiency of the SR-TENG was evaluated at different wind speeds, as shown in Table S2. However, as the self-regulating function necessitates a relatively large structural footprint, the volumetric power density of the SR-TENG is comparatively lower than that of some state-of-the-art wind energy harvesters. A conversion efficiency of 0.0619% was achieved at a wind speed of 3.7 m/s. The alternating voltage generated by the TENG is converted into a direct current signal following rectification. This signal is characterized by the manifestation of substantial ripple, rendering it unsuitable for direct supply to downstream circuits. Consequently, a storage capacitor needs to be incorporated after the rectifier circuit. In order to investigate the effects of storage capacitance and load resistance on output characteristics, a selection of capacitors with a range of storage capacities, specifically 1 μF, 2.2 μF, 4.7 μF, and 10 μF, were chosen and tested with loads of 1 MΩ, 10 MΩ, and 100 MΩ, respectively. As illustrated in Figure 3c–e, at equivalent load resistances, smaller capacitors demonstrate substantially faster voltage rise rates in comparison to their larger counterparts. This phenomenon can be attributed to the enhanced efficiency of charging and discharging processes in smaller capacitors. Furthermore, output voltage tests were conducted across loads ranging from 1 MΩ to 100 MΩ under a constant 47 μF storage capacitor, as illustrated in Figure 3f. When stored capacitance conditions are constant, an increase in load resistance also impacts the voltage rise time. Due to the high internal equivalent impedance of the TENG itself, as the external load resistance increases, the total equivalent impedance of the charging circuit increases, significantly limiting the instantaneous charging current. At the same time, the nonlinear conduction characteristics of the rectifier diode lead to a reduction in the effective conduction angle during each cycle, further decreasing the average charging current and thus slowing down the voltage rise rate.

### 3.3. Design and Optimization of Power Management Circuit

**Figure 4:**
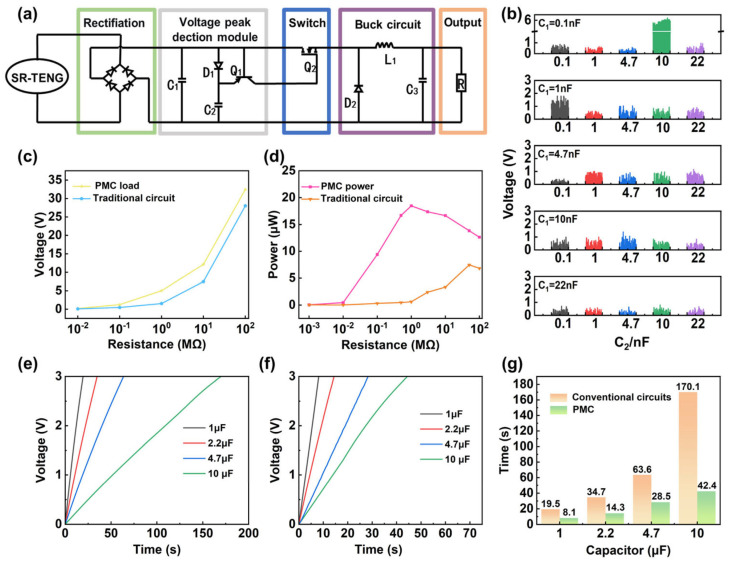
Power management characteristics of the SR-TENG: (**a**) Design framework of the PMC. (**b**) Output voltage signal of the peak detection module. (**c**) Comparison of output voltages. (**d**) Comparison of output power. (**e**) Voltage curve of the SR-TENG directly charging the capacitor. (**f**) Voltage curve of the SR-TENG charging the capacitor through the PMC. (**g**) Comparison of charging performance.

The charging rate of storage capacitors by the SR-TENG is severely constrained by its high internal impedance and the large voltage fluctuations inherent in direct rectification, which lead to a sluggish voltage increase. To overcome these limitations, a power management circuit (PMC) was designed and optimized to achieve efficient integration, regulation, and utilization of the generated energy, thereby significantly improving both energy harvesting efficiency and output stability. The PMC consists of a rectification bridge, a voltage peak-detection module, a switching module, a buck converter, and an output module (Figure 4a). Through the PMC, the AC output generated by the SR-TENG is first rectified into DC, simultaneously charging the energy storage capacitor (C_1_) and the detection capacitor (C_2_). When the voltage across C_2_ exceeds that of C_1_, transistor Q_1_ turns on, subsequently driving MOSFET Q_2_ to conduct. At this moment, the energy accumulated in C_1_ is transferred to the load via the buck circuit (composed of L_1_ and C_3_), achieving concentrated energy release and a regulated output. The voltage peak-detection and switching modules are the core components of this system. During the operation of the SR-TENG, both C_1_ and C_2_ are charged simultaneously. Due to the high internal impedance of the TENG, the capacitor voltages tend to saturate over time. However, the detection branch involving C_2_ and transistor Q_1_ exhibits a lower equivalent impedance, leading to a faster voltage response. Once the voltage of C_2_ surpasses that of C_1_, the emitter voltage of Q_1_ exceeds its base voltage, triggering conduction and activating MOSFET Q_2_ to release the stored energy to the load.

To verify the response characteristics of the peak-detection module, we systematically evaluated the effects of the storage (C_1_) and detection (C_2_) capacitors. With a 1 MΩ resistor connected as the load to monitor voltage signals, various capacitance combinations (0.1 nF to 22 nF) were examined (Figure 4b). Following an analysis of pulse amplitude and frequency, C_1_ = 0.1 nF and C_2_ = 10 nF were identified as the optimal parameters. A comparative analysis of the output performance against a traditional rectification circuit (Figure 4c) reveals that, while voltage increases with load resistance in both cases, the PMC yields consistently superior output voltages. This advantage is especially distinct at resistances exceeding 10 MΩ. Consequently, the PMC substantially boosts the energy harvesting efficiency of the SR-TENG. As depicted in Figure 4d, the effective output power sees a remarkable improvement—specifically, a 30.39-fold increase at a 1 MΩ load. Crucially, the optimal impedance matching range is shifted downwards, making it far more compatible with the actual impedance of typical electronics. The charging capability is further detailed in Figure 4e,f, which compare the time required to charge capacitors (1 μF to 10 μF) to 3 V under direct charging and PMC conditions. In contrast to the sluggish voltage rise in the traditional circuit, the PMC enables a rapid voltage increase and drastically reduced charging times. As quantified in Figure 4g, the PMC accelerates the charging of a 10 μF capacitor to 3 V by four times compared to its counterpart.

### 3.4. SR-TENG for Self-Powered Wireless Sensing

**Figure 5:**
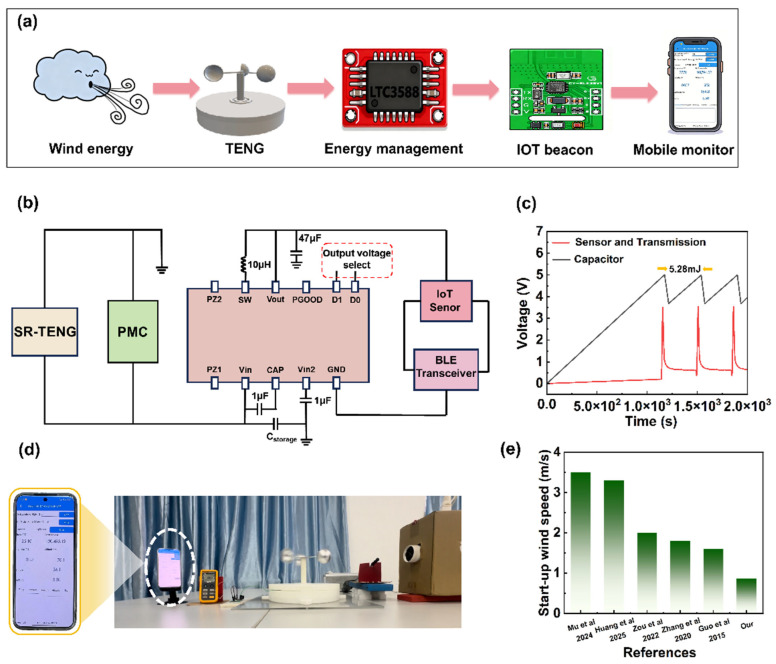
Demonstration of the SR-TENG for powering wireless sensing electronics: (**a**) Working process of the self-powered system based on SR-TENG for wireless temperature-humidity monitoring. (**b**) Circuit diagram for energy management and wireless data transmission. (**c**) Voltage profile of a 1 mF capacitor powering the wireless temperature-humidity sensor. (**d**) Photograph showing the real-time wireless temperature-humidity monitoring powered by the SR-TENG. (**e**) Start-up wind speed of this work compared with previous work [1,28,41,50,51].

Featuring a self-adaptive mode-switching mechanism governed by wind speed, the SR-TENG demonstrates exceptional adaptability across a broad range of wind velocities, allowing for flexible deployment in diverse natural environments. When integrated with a PMC, this system effectively harvests ambient wind energy and efficiently converts it into electricity to power wireless sensing electronics. As a proof-of-concept, we developed a self-powered environmental monitoring system (Figure 5a) that integrates the SR-TENG, a PMC, temperature-humidity sensors, and a wireless transmission unit. The entire system operates solely on the wind energy harvested by the SR-TENG, eliminating the need for external power sources. Ultimately, the collected data is transmitted in real-time to mobile devices via an IoT terminal.

The power supply process of the entire system driven by the SR-TENG is illustrated in Figure 5b. Initially, the AC output generated by the SR-TENG is rectified via the PMC and stored in a 1 mF capacitor. Subsequently, the stored energy is regulated by an LTC3588 module to provide a stable 3.3 V DC output, powering the low-power GY-BLE39 IoT sensor node. Figure 5c shows the voltage variation curve of the energy storage capacitor and the wireless sensor node. The experimental results indicate that, at a wind speed of 8 m/s, the initial charging phase of the energy storage capacitor to 5 V takes approximately 1140 s. During this phase, the LTC3588 does not trigger the output, and the GY-BLE39 sensor remains in standby mode. Once the capacitor voltage reaches 5.0 V, the LTC3588 triggers the output and provides stable 3.3 V power to the GY-BLE39. The sensor consumes energy while performing temperature and humidity data collection and Bluetooth wireless transmission tasks, resulting in a significant voltage drop of the energy storage capacitor from 5.0 V to 3.8 V. The system then enters a recharging cycle. According to Note S3, the single energy consumption is 5.28 mJ. After approximately 360 s, the capacitor is recharged to 5 V, and the sensor system resumes operation. Furthermore, Figure 5d and Video S1 demonstrate the SR-TENG’s capability to start up at a low wind speed of 0.86 m/s. We investigated the performance of TENGs in previous studies and compared the SR-TENG with them (Figure 5e [1,28,41,50,51]), which demonstrates that the SR-TENG exhibits a significant advantage in terms of starting wind speed. Moreover, real-time wireless monitoring of temperature and humidity was realized at a wind speed of 8 m/s.

## 4. Conclusions

In summary, we have proposed a self-regulating SR-TENG featuring wind speed adaptive mode switching. Through the synergistic interaction of centrifugal, elastic, and friction forces, the device achieves a low start-up wind speed of 0.86 m/s. It operates in a non-contact mode at low wind speeds and automatically transitions to a contact mode at high wind speeds, thereby further enhancing output performance. Furthermore, when coupled with a PMC, the energy harvesting and storage efficiency of the SR-TENG are significantly improved. Specifically, the effective output power across a 1 MΩ load is boosted by 30.39 times, and the charging rate for a 10 μF capacitor is increased by 4 times. As a proof of concept, a smart platform integrating the SR-TENG, PMC, and a wireless sensing node was constructed. By harvesting ambient wind energy, this self-powered system successfully realizes real-time tracking of environmental temperature and humidity. This integrated solution not only eliminates the reliance on external power supplies but also demonstrates immense potential for distributed environmental monitoring in the IoT. In summary, we have proposed a self-regulating SR-TENG featuring wind speed adaptive mode switching. Through the synergistic interaction of centrifugal, elastic, and friction forces, the device achieves a low start-up wind speed of 0.86 m/s. It operates in a non-contact mode at low wind speeds and automatically transitions to a contact mode at high wind speeds, thereby further enhancing output performance. Systematic structural optimization of spring specifications and electrode pair quantity enables the SR-TENG to reach a peak open-circuit voltage of 140 V, a short-circuit current of 12.5 μA, and a transferred charge of 300 nC at 375 rpm, and the device maintains excellent durability with no observable performance degradation during 23,000 s of continuous operation at this rotational speed. Furthermore, when coupled with a PMC, the energy harvesting and storage efficiency of the SR-TENG are significantly improved. Specifically, the effective output power across a 1 MΩ load is boosted by 30.39 times, and the charging rate for a 10 μF capacitor is increased by 4 times. As a proof of concept, a smart platform integrating the SR-TENG, PMC, and a wireless sensing node was constructed. By harvesting ambient wind energy, this self-powered system successfully realizes real-time tracking of environmental temperature and humidity. This integrated solution not only eliminates the reliance on external power supplies but also demonstrates immense potential for distributed environmental monitoring, self-powered wireless sensing and smart transportation systems in the IoT.

## Data Availability

The original contributions presented in this study are included in the article/Supplementary Materials. Further inquiries can be directed to the corresponding author.

## Supplementary Materials

The following supporting information can be downloaded at: https://www.mdpi.com/article/10.3390/mi17030373/s1, Figure S1: Photo of SR-TENG. (a) Slider, (b) rotor, (c) stator, and (d) SR-TENG; Figure S2. Influence of spring diameter and length on the output performance of the SR-TENG. (a–c) Peak voltage, current, and transferred charge of different springs at 75 rpm, (d–f) Peak voltage, current, and transferred charge of different springs at 150 rpm, (g–i) Peak voltage, current, and transferred charge of different springs at 225 rpm, (j–l) Peak voltage, current, and transferred charge of different springs at 300 rpm, (m–o) Peak voltage, current, and transferred charge of different springs at 375 rpm, (p–r) Peak voltage, current, and transferred charge of different springs at 450 rpm; Figure S3. Force analysis under non-contact mode and contact mode; Figure S4. Influence of the electrode number on the SR-TENG output. (a–c) Peak voltage, current and transferred charge with different numbers of electrodes at 75 rpm, (d–f) Peak voltage, current and transferred charge with different numbers of electrodes at 150 rpm, (g–i) Peak voltage, current and transferred charge with different numbers of electrodes at 225 rpm, (j–l) Peak voltage, current and transferred charge with different numbers of electrodes at 300 rpm, (m–o) Peak voltage, current and transferred charge with different numbers of electrodes at 375 rpm, (p–r) Peak voltage, current and transferred charge with different numbers of electrodes at 450 rpm; Figure S5. Output Voltage at Varying Motor Speeds. (a) Output voltage when the motor speed increases from 75 rpm to 150 rpm, (b) Output voltage when the motor speed decreases from 150 rpm to 75 rpm; Figure S6. Output performance of SR-TENG at different wind speeds. (a) Open-circuit voltage of SR-TENG at different wind speeds. (b) Short-circuit current of SR-TENG at different wind speeds; Figure S7. Schematic diagram of the relationship among wind speed, rotational speed and output current. (a) Fitting curve of motor rotational speed versus output current. (b) Fitting curve of wind speed versus output current; Figure S8. Output power of SR-TENG as a function of load resistance at different rotational speeds; Table S1. Comparison of the performance between the SR-TENG and previously studied TENGs; Table S2. Power conversion efficiency at different wind speeds; Note S1. The formula for the spring stiffness k; Note S2. The formula for the critical rotation speed (nth); Note S3. The formula for capacitor energy change; Video S1: The real-time wireless temperature-humidity monitoring powered by the SR-TENG.

## References

[1] Guo H., Chen J., Yeh M.-H., Fan X., Wen Z., Li Z., Hu C., Wang Z.L. (2015). An Ultrarobust High-Performance Triboelectric Nanogenerator Based on Charge Replenishment. ACS Nano.

[2] Liu D., Luo J., Huang L., Chen M., Ji M., Wang Z.L., Kang J. (2025). Triboelectric nanogenerators as a practical approach for wind energy harvesting: Mechanisms, designs, and applications. Nano Energy.

[3] Zhang B., He L., Zhang R., Yuan W., Wang J., Hu Y., Zhao Z., Zhou L., Wang J., Wang Z.L. (2023). Achieving Material and Energy Dual Circulations of Spent Lithium-Ion Batteries via Triboelectric Nanogenerator. Adv. Energy Mater..

[4] Zhang B., He L., Wang J., Liu Y., Xue X., He S., Zhang C., Zhao Z., Zhou L., Wang J. (2023). Self-powered recycling of spent lithium iron phosphate batteries via triboelectric nanogenerator. Energy Environ. Sci..

[5] Dong K., Peng X., An J., Wang A.C., Luo J., Sun B., Wang J., Wang Z.L. (2020). Shape adaptable and highly resilient 3D braided triboelectric nanogenerators as e-textiles for power and sensing. Nat. Commun..

[6] Shi Q., Sun Z., Zhang Z., Lee C. (2021). Triboelectric Nanogenerators and Hybridized Systems for Enabling Next-Generation IoT Applications. Research.

[7] Portilla L., Loganathan K., Faber H., Eid A., Hester J.G.D., Tentzeris M.M., Fattori M., Cantatore E., Jiang C., Nathan A. (2022). Wirelessly powered large-area electronics for the Internet of Things. Nat. Electron..

[8] Luo B., Will-Cole A.R., Dong C., He Y., Liu X., Lin H., Huang R., Shi X., Mcconney M., Page M. (2024). Magnetoelectric microelectromechanical and nanoelectromechanical systems for the IoT. Nat. Rev. Electr. Eng..

[9] Yan X., Jin Y., Chen X., Zhang C., Hao C., Wang Z. (2020). Nature-inspired surface topography: Design and function. Sci. China Phys. Mech. Astron..

[10] He Q., Lee M., Wu W. (2025). Fully integrated magneto-mechano-triboelectric nanogenerator for power-line stray magnetic field harvesting with record 48.2 mW/cm^3^ packaged power density. Nano Energy.

[11] Zhao B., Wang Y., Huang S., Tian T., Liao X., Wang W., Li Z. (2025). High-durability pendulum-structured TENG-EMG hybrid with stacked liquid-solid triboelectric layers for efficient low-frequency ocean wave energy capture. Chem. Eng. J..

[12] Li H., Wang J., Liang L., Hou S. (2025). Design of an efficient blue energy harvesting system based on triboelectric nanogenerators with frequency upconversion and networking strategies. Nano Energy.

[13] Guo W., Long Y., Bai Z., Wang X., Liu H., Guo Z., Tan S., Guo H., Wang Y., Miao Y. (2022). Variable stiffness triboelectric nano-generator to harvest high-speed railway bridge’s vibration energy. Energy Conv. Manag..

[14] Cao L.N.Y., Su E., Xu Z., Wang Z.L. (2023). Fully enclosed microbeads structured TENG arrays for omnidirectional wind energy harvesting with a portable galloping oscillator. Mater. Today.

[15] Tan D., Ou X., Jia Z., Zhou J., Wang K., Sun H. (2025). A cylindrical bistable hybrid triboelectric-electromagnetic energy harvester for harvesting low-frequency vibration energy. Energy.

[16] Sagar P., Sinha N., Shukla M., Yadav T., Kumar B. (2025). Flexible piezoelectric nanogenerator based on Nd-ZnS nanoplates for human body movements detection and wearable electronics. J. Alloys Compd..

[17] Zhu M., Yi Z., Yang B., Lee C. (2021). Making use of nanoenergy from human—Nanogenerator and self-powered sensor enabled sustainable wireless IoT sensory systems. Nano Today.

[18] Zhou N., Hou Z., Zhang Y., Cao J., Bowen C.R. (2021). Enhanced swing electromagnetic energy harvesting from human motion. Energy.

[19] Liao W., Su X., Li B., Li H., Zhang K., Fang F., Huang X. (2025). Mimosa-inspired tribo-electromagnetic nanogenerator for adaptive wind energy harvesting and intelligent wind monitoring. Nano Energy.

[20] Shu L., Fang L., Wang F., Li Z., Guo Y., Zhang H., Wang Z., He W., Rasheed A., Fan K. (2025). Wind speed adaptive triboelectric nanogenerator with low start-up wind speed, enhanced durability and high power density via the synergistic mechanism of magnetic and centrifugal forces for intelligent street lamp system. Nano Energy.

[21] Han H., Luo J., Gao L., Deng Q., Han L., Zhao L., Hu N., Mu X. (2025). A triboelectric nanogenerator with efficiency enhancement based on relative inversion for smart agriculture. Nano Mater. Sci..

[22] Xiong T., Xu Z., He Q., Li S., Tang G., Meng Y., Zhang X., Liu C., Shi C., Wang Z.L. (2025). Direction-adaptive triboelectric-electromagnetic hybrid nanogenerator for harvesting omnidirectional breeze wind energy. Nano Energy.

[23] Wang X., Tang M., Han Y., Tairab A.M., Yu J., Han L., Zhang Z., Kong L. (2023). An omnidirectional hybrid wind-wave energy harvester based on a coaxial contra-rotation mechanism for unmanned surface vessels. Energy Conv. Manag..

[24] Hu Y., Li X., Gao Y., Zhao Z., Liu X., He L., Zhang B., Zhou L., Wang Z.L., Wang J. (2024). A Combined Wind Harvesting and Speed Sensing System Based on Constant-Voltage Triboelectric Nanogenerator. Adv. Energy Mater..

[25] Ren Z., Wang Z., Liu Z., Wang L., Guo H., Li L., Li S., Chen X., Tang W., Wang Z.L. (2020). Energy Harvesting from Breeze Wind (0.7–6 m s^−1^) Using Ultra-Stretchable Triboelectric Nanogenerator. Adv. Energy Mater..

[26] Zhang X., Yu Y., Xia X., Zhang W., Cheng X., Li H., Wang Z.L., Cheng T. (2023). Multi-Mode Vibrational Triboelectric Nanogenerator for Broadband Energy Harvesting and Utilization in Smart Transmission Lines. Adv. Energy Mater..

[27] Zhao X., Nashalian A., Ock I.W., Popoli S., Xu J., Yin J., Tat T., Libanori A., Chen G., Zhou Y. (2022). A Soft Magnetoelastic Generator for Wind-Energy Harvesting. Adv. Mater..

[28] Zhang Y., Zeng Q., Wu Y., Wu J., Yuan S., Tan D., Hu C., Wang X. (2020). An Ultra-Durable Windmill-Like Hybrid Nanogenerator for Steady and Efficient Harvesting of Low-Speed Wind Energy. Nano-Micro Lett..

[29] Ma G., Gao F., Zhang M., Wang Y., Gu C., Meng F., She J., Song Y., He X., Wang D. (2024). An Endurable Triboelectric Nanogenerator for Wind Energy Harvesting Based on Centrifugal Force Induced Automatic Switching between Sliding and Rolling Modes. ACS Sustain. Chem. Eng..

[30] Ba K., Liu G., Ma G., Chen C., Pu L., He X., Chen X., Wang Y., Zhu Q., Wang D. (2024). Bionic perception and transmission neural device based on a self-powered concept. Cell Rep. Phys. Sci..

[31] Teli A., Bhatta T., Sharma S., Pradhan G.B., Sapkota S., Jo M.S., Park J.Y. (2025). Magnetic repulsion-assisted hybrid breeze wind energy harvester with non-contact triboelectric sensor for self-sustainable device conditioning and environmental monitoring applications. Nano Energy.

[32] Ma G., Li B., Niu S., Zhang J., Wang D., Wang Z., Zhou L., Liu Q., Liu L., Wang J. (2022). A bioinspired triboelectric nanogenerator for all state energy harvester and self-powered rotating monitor. Nano Energy.

[33] Ma G., Wang D., Wang J., Li J., Wang Z., Li B., Mu Z., Niu S., Zhang J., Ba K. (2023). A durable triboelectric nanogenerator with a coaxial counter-rotating design for efficient harvesting of random mechanical energy. Nano Energy.

[34] Long L., Liu W., Wang Z., He W., Li G., Tang Q., Guo H., Pu X., Liu Y., Hu C. (2021). High performance floating self-excited sliding triboelectric nanogenerator for micro mechanical energy harvesting. Nat. Commun..

[35] Yuan S., Zeng Q., Tan D., Luo Y., Zhang X., Guo H., Wang X., Wang Z.L. (2022). Scavenging breeze wind energy (1–8.1 m s^−1^) by minimalist triboelectric nanogenerator based on the wake galloping phenomenon. Nano Energy.

[36] Li H., Wen J., Ou Z., Su E., Xing F., Yang Y., Sun Y., Wang Z.L., Chen B. (2023). Leaf-Like TENGs for Harvesting Gentle Wind Energy at An Air Velocity as Low as 0.2 m s^−1^. Adv. Funct. Mater..

[37] Xu Z., Chen L., Zhang Z., Han J., Chen P., Hong Z., Jiang T., Wang Z.L. (2024). Durable Roller-Based Swing-Structured Triboelectric Nanogenerator for Water Wave Energy Harvesting. Small.

[38] Bai Y., Xu L., Lin S., Luo J., Qin H., Han K., Wang Z.L. (2020). Charge Pumping Strategy for Rotation and Sliding Type Triboelectric Nanogenerators. Adv. Energy Mater..

[39] Liu S., Li X., Wang Y., Yang Y., Meng L., Cheng T., Wang Z.L. (2021). Magnetic switch structured triboelectric nanogenerator for continuous and regular harvesting of wind energy. Nano Energy.

[40] Yong S., Wang J., Yang L., Wang H., Luo H., Liao R., Wang Z.L. (2021). Auto-Switching Self-Powered System for Efficient Broad-Band Wind Energy Harvesting Based on Dual-Rotation Shaft Triboelectric Nanogenerator. Adv. Energy Mater..

[41] Zou H., Zhao L., Wang Q., Gao Q., Yan G., Wei K., Zhang W. (2022). A self-regulation strategy for triboelectric nanogenerator and self-powered wind-speed sensor. Nano Energy.

[42] Chen J., Guo H., Hu C., Wang Z.L. (2020). Robust Triboelectric Nanogenerator Achieved by Centrifugal Force Induced Automatic Working Mode Transition. Adv. Energy Mater..

[43] Fu S., He W., Tang Q., Wang Z., Liu W., Li Q., Shan C., Long L., Hu C., Liu H. (2022). An Ultrarobust and High-Performance Rotational Hydrodynamic Triboelectric Nanogenerator Enabled by Automatic Mode Switching and Charge Excitation. Adv. Mater..

[44] Min G., Xu Y., Cochran P., Gadegaard N., Mulvihill D.M., Dahiya R. (2021). Origin of the contact force-dependent response of triboelectric nanogenerators. Nano Energy.

[45] Jin C., Kia D.S., Jones M., Towfighian S. (2016). On the contact behavior of micro-/nano-structured interface used in vertical-contact-mode triboelectric nanogenerators. Nano Energy.

[46] Qin Q., Cao X., Wang N. (2023). Ball-Mill-Inspired Durable Triboelectric Nanogenerator for Wind Energy Collecting and Speed Monitoring. Nanomaterials.

[47] Yang J., Chen J., Su Y., Jing Q., Li Z., Yi F., Wen X., Wang Z., Wang Z.L. (2015). Eardrum-Inspired Active Sensors for Self-Powered Cardiovascular System Characterization and Throat-Attached Anti-Interference Voice Recognition. Adv. Mater..

[48] Yi F., Lin L., Niu S., Yang P.K., Wang Z., Chen J., Zhou Y., Zi Y., Wang J., Liao Q. (2015). Stretchable-Rubber-Based Triboelectric Nanogenerator and Its Application as Self-Powered Body Motion Sensors. Adv. Funct. Mater..

[49] Zhang C., Liu Y., Zhang B., Yang O., Yuan W., He L., Wei X., Wang J., Wang Z.L. (2021). Harvesting Wind Energy by a Triboelectric Nanogenerator for an Intelligent High-Speed Train System. ACS Energy Lett..

[50] Mu Q., He W., Shan C., Fu S., Du S., Wang J., Wang Z., Li K., Hu C. (2024). Achieving High-Efficiency Wind EnergyHarvesting Triboelectric Nanogenerator by Coupling Soft Contact, Charge Space Accumulation, and Charge Dissipation Design. Adv. Funct. Mater..

[51] Huang X., Hu D., Wang Q., Wu Z., Wang N., Chen Z., Xu S., Chi M., Chen S. (2025). Hybrid Nanogenerator Harvesting Electric-Field and Wind Energy for Self-Powered Sensors on High-Voltage Transmission Lines. Adv. Funct. Mater..

