# Supplementary material for: Self-Regulating Wind Speed Adaptive Mode Switching for Efficient Wind Energy Harvesting Towards Self-Powered Wireless Sensing
Source: Micromachines (Basel). 2026 Mar 19;17(3):373. doi: 10.3390/mi17030373 (PMC13029519; doi:10.3390/mi17030373)
Supplement: Supplementary file 1 [file micromachines-17-00373-s001.zip › Supplementary material-edited.pdf]

Self-Regulating Wind Speed Adaptive Mode Switching for Efficient Wind Energy Harvesting Towards Self-Powered Wireless Sensing

Ruifeng Li [1], Chenming Wang [1], Yiao Pan [1], Jianhua Zeng [1,*], Youchao Qi [2,*], Ping Zhang [1,*]

[1] School of Mechanical and Electrical Engineering, Guilin University of Electronic Technology, No. 1 Jinji Road, Guilin, Guangxi, 541004, P. R. China

[2] CAS Center for Excellence in Nanoscience, Beijing Key Laboratory of Micro-nano Energy and Sensor, Beijing Institute of Nanoenergy and Nanosystems, Chinese Academy of Sciences, Beijing 101400, P. R. China

[3] School of Fashion and Textiles, The Hong Kong Polytechnic University, Hong Kong SAR, China

Corresponding author: zengjianhua@guet.edu.cn (Jianhua Zeng), qiyouchaoo@163.com (Youchao Qi), pingzhang@guet.edu.cn (Ping Zhang)

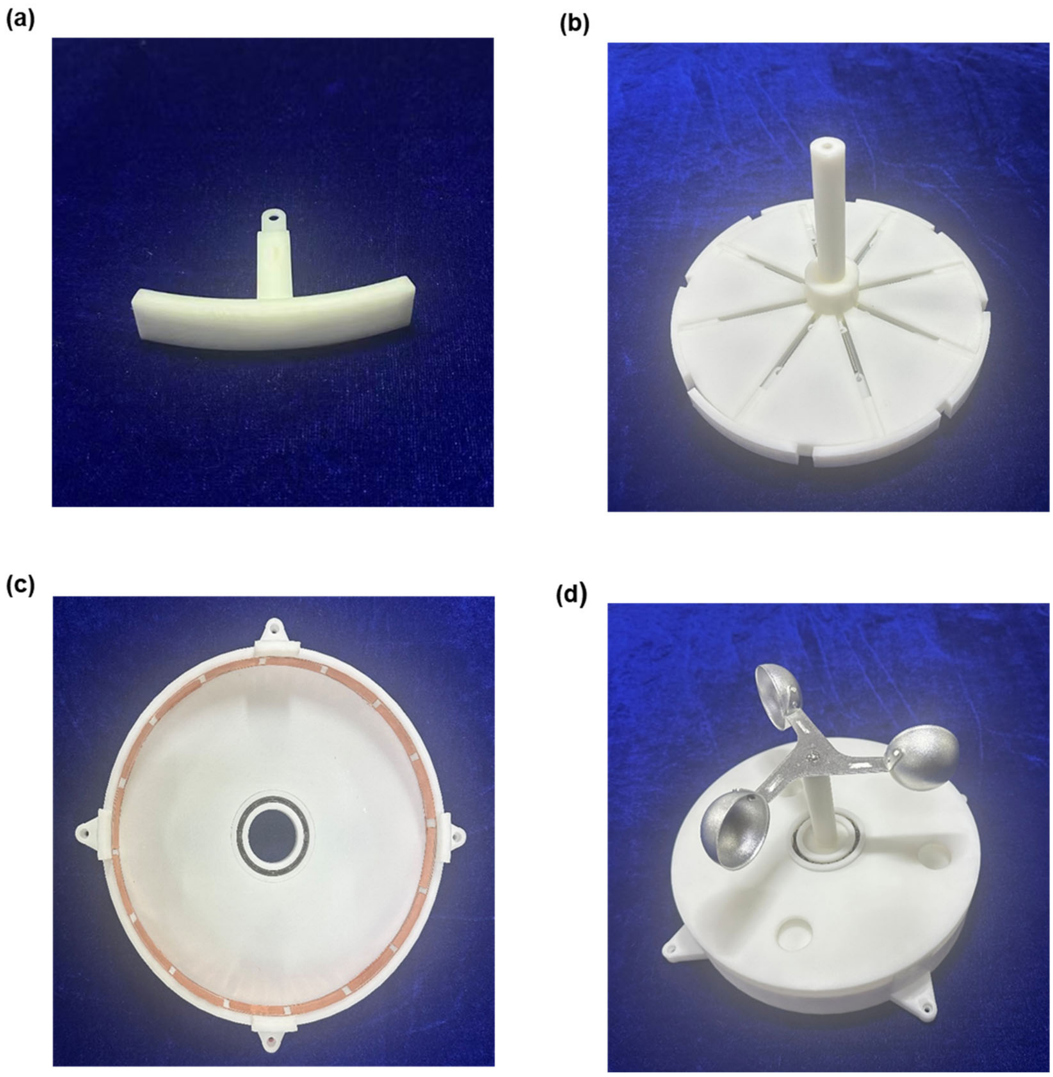

Figure S1. Photo of SR-TENG: (a) slider, (b) rotor, (c) stator, and (d) SR-TENG.

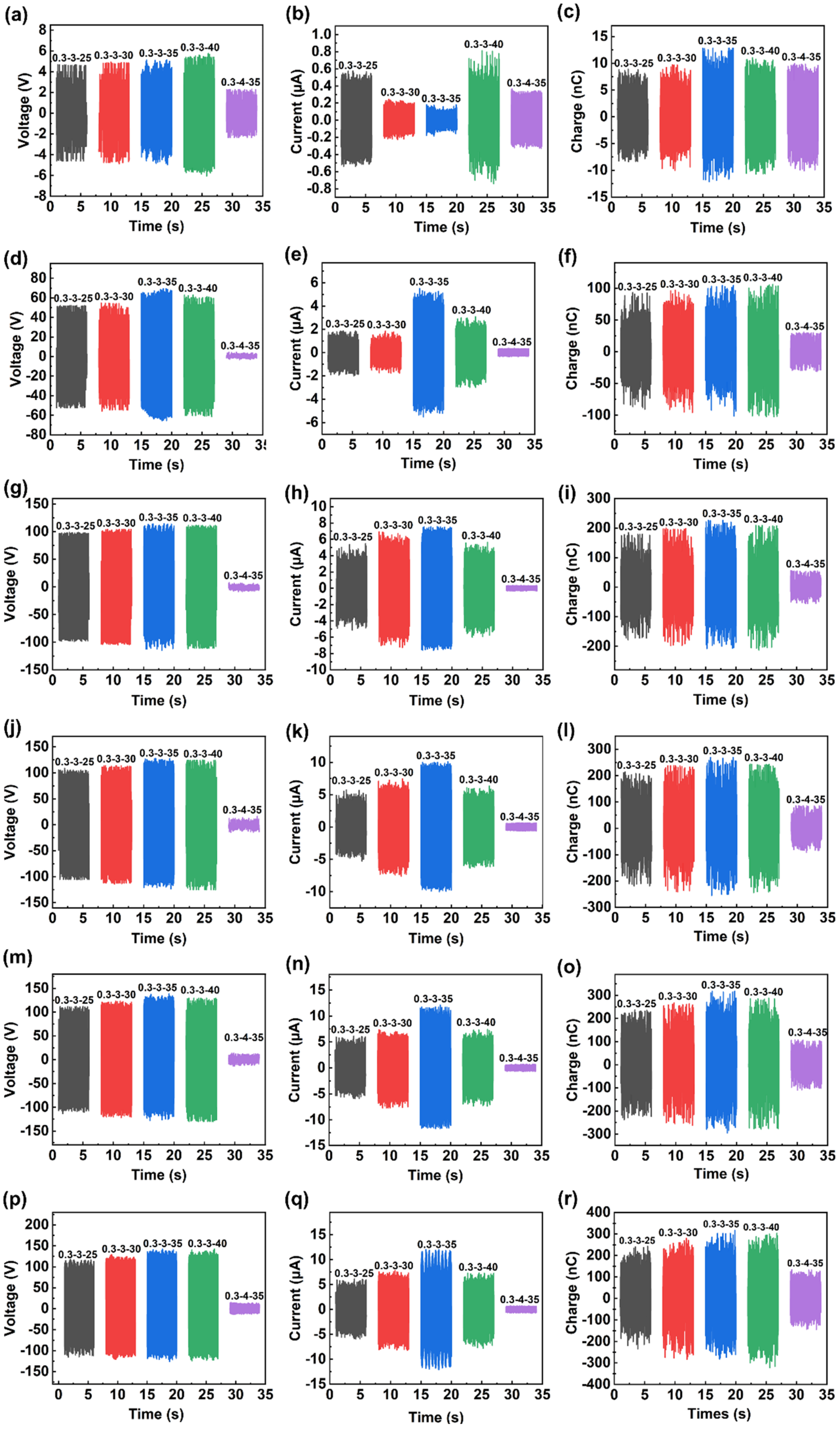

(a)
(b)
(c)
(d)
(e)
(f)
(g)
(h)
(i)
(j)
(k)
(l)
(m)
(n)
(o)
(p)
(q)
(r)
Voltage (V)
Current (μA)
Charge (nC)
Time (s)
Times (s)
0.3-3-25
0.3-3-30
0.3-3-35
0.3-3-40
0.3-4-35

Figure S2. Influence of spring diameter and length on the output performance of the SR-TENG: (a-c) Peak voltage, current, and transferred charge of different springs at 75 rpm. (d-f) Peak voltage, current, and transferred charge of different springs at 150 rpm. (g-i) Peak voltage, current, and transferred charge of different springs at 225 rpm. (j-l) Peak voltage, current, and transferred charge of different springs at 300 rpm. (m-o) Peak voltage, current, and transferred charge of different springs at 375 rpm. (p-r) Peak voltage, current, and transferred charge of different springs at 450 rpm.

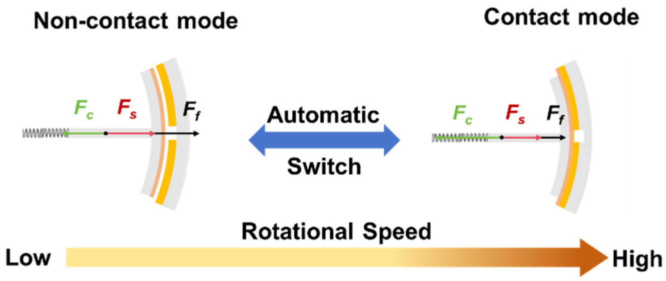

Figure S3. Force analysis under non-contact mode and contact mode.

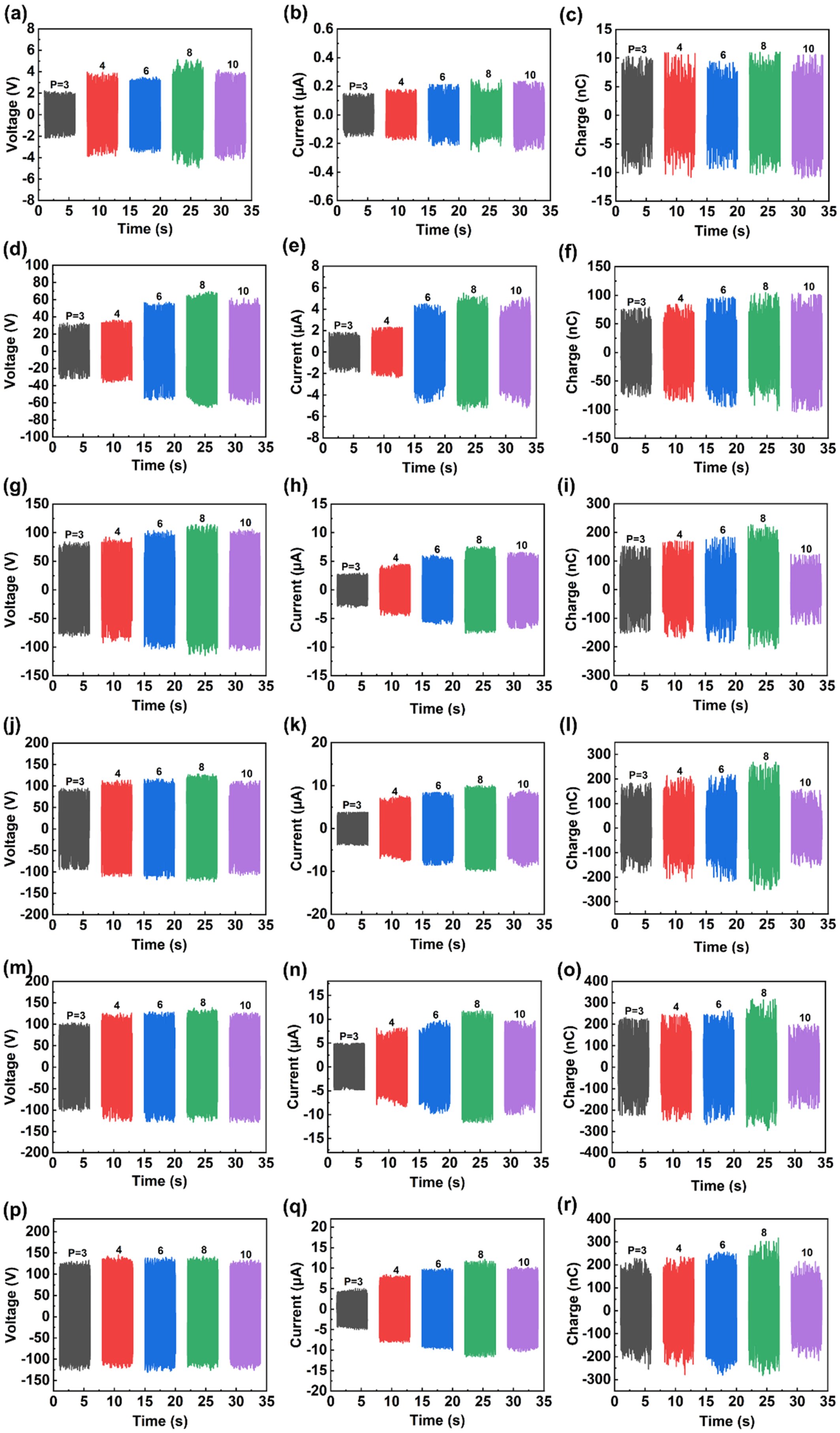
(a)
(b)
(c)
(d)
(e)
(f)
(g)
(h)
(i)
(j)
(k)
(l)
(m)
(n)
(o)
(p)
(q)
(r)
Voltage (V)
Current (μA)
Charge (nC)
Time (s)
P=3
4
6
8
10

Figure S4. Influence of the electrode number on the SR-TENG output: (a-c) Peak voltage, current and transferred charge with different numbers of electrodes at 75 rpm. (d-f) Peak voltage, current and transferred charge with different numbers of electrodes at 150 rpm. (g-i) Peak voltage, current and transferred charge with different numbers of electrodes at 225 rpm. (j-l) Peak voltage, current and transferred charge with different numbers of electrodes at 300 rpm. (m-o) Peak voltage, current and transferred charge with different numbers of electrodes at 375 rpm. (p-r) Peak voltage, current and transferred charge with different numbers of electrodes at 450 rpm.

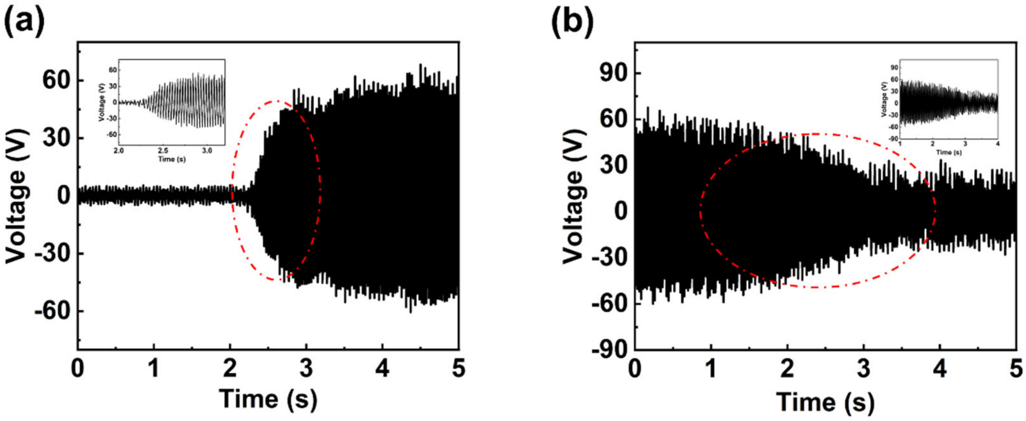

Figure S5. Output voltage at varying motor speeds: (a) Output voltage when the motor speed increases from 75 rpm to 150 rpm. (b) Output voltage when the motor speed decreases from 150 rpm to 75 rpm.

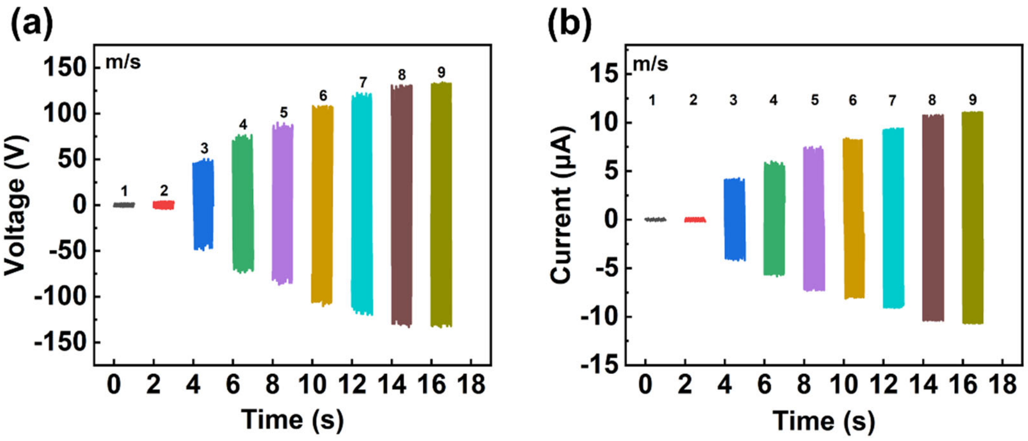

Figure S6. Output performance of SR-TENG at different wind speeds: (a) Open-circuit voltage of SR-TENG at different wind speeds. (b) Short-circuit current of SR-TENG at different wind speeds.

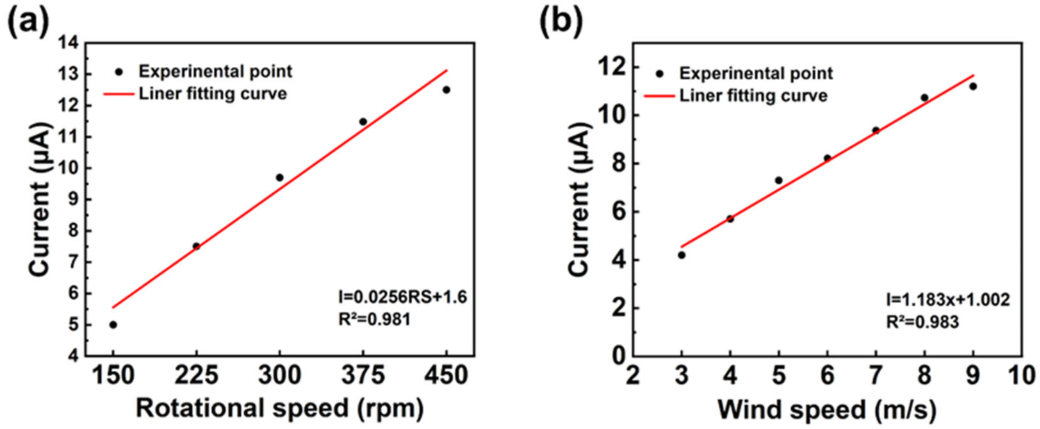

Figure S7. Schematic diagram of the relationship among wind speed, rotational speed and output current: (a) Fitting curve of motor rotational speed versus output current. (b) Fitting curve of wind speed versus output current.

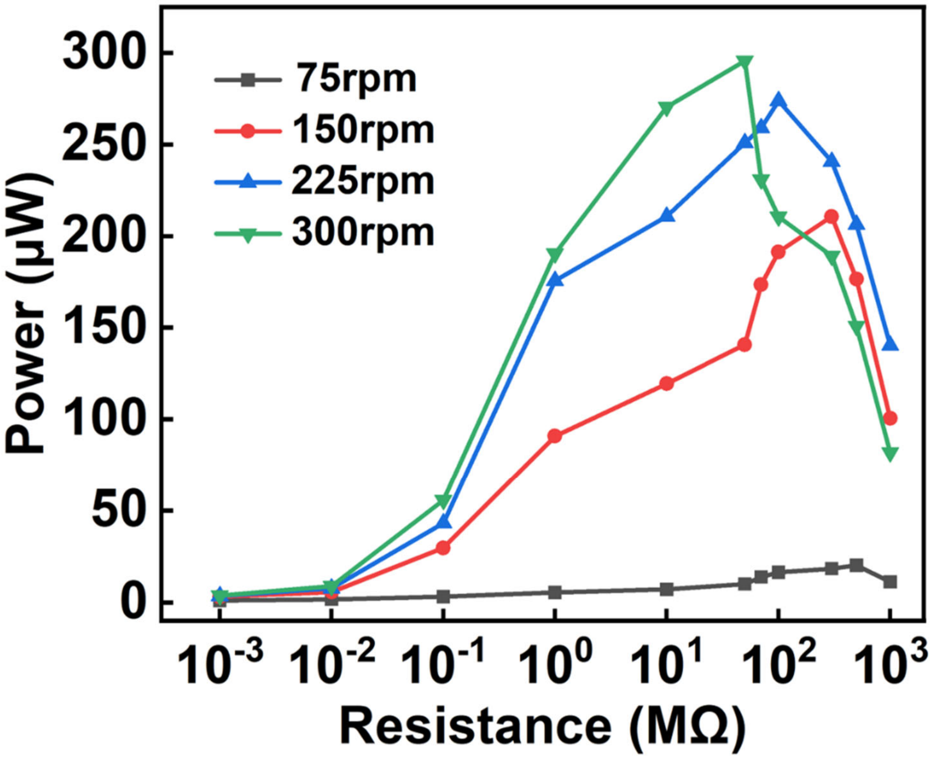

Figure S8. Output power of SR-TENG as a function of load resistance at different rotational speeds.

Table S1. Comparison of the performance between the SR-TENG and previously studied TENGs.

Name	Max voltage outputs	Durability	Attenuation of electrical energy	Power	Reference
BMI-TENG	257V	5000	19.87%	685μW	Ref1
BMS	38V	40000	basically unchanged	/	Ref2
SR-based-TENG	65V	5000	basically unchanged	/	Ref3
CEMA-TENG	240V	72000	6%	18.8mW	Ref4
ER-TENG	600V	250000	20%	29.21mW	Ref5
SM-TENG	130V	550000	basically unchanged	2.45mW	Ref6
SR-TENG	140V	143000	basically unchanged	320μW	This work

Table S2. Power conversion efficiency at different wind speeds.

Wind Speed	Peak Power	Average Power	Conversion Efficiency
2.1m/s	20μW	25μW	0.0441%
3.7 m/s	191μW	192μW	0.0619%
5.4 m/s	238μW	244μW	0.0253%
7 m/s	296μW	301μW	0.0143%
8.6 m/s	320μW	655μW	0.0168%

Note S1. The formula for the spring stiffness *k*.

The formula for spring stiffness k is:

$$k = \frac{Gd^4}{8D^3 n}$$

where d is the diameter of the spring wire, G is the shear modulus of the material, D is the mean diameter of the spring, and n is the number of active coils.

Note S2. The formula for the critical rotation speed (n_{th}).

The expression for the critical rotation speed is:

$$n_{th} = \frac{60}{2\pi}\sqrt{\frac{k \times g}{m\left(R_0 + g\right)}}$$

where k is the spring stiffness, g is the initial gap, m is the mass of the slider and R_0 is the initial radius of the slider's center of mass.

Note S3. The formula for capacitor energy change.

The expression for energy:

$$\Delta E = \frac{1}{2} C (V_{\max}^2 - V_{\min}^2)$$

where *ΔE* is the change in the electric field energy stored in the capacitor, *C* is the capacitance of the capacitor, *Vmax* is the maximum voltage to which the capacitor is charged, and *Vmin* is the minimum voltage of the capacitor after discharge.

Reference

1. Qin, Q.; Cao, X.; Wang, N. Ball-Mill-Inspired Durable Triboelectric Nanogenerator for Wind Energy Collecting and Speed Monitoring. Nanomaterials 2023, 13, 939, doi: 10.3390/nano13050939.
2. Yang, J.; Chen, J.; Su, Y.; Jing, Q.; Li, Z.; Yi, F.; Wen, X.; Wang, Z.; Wang, Z.L. Eardrum-Inspired Active Sensors for Self-Powered Cardiovascular System Characterization and Throat-Attached Anti-Interference Voice Recognition. Adv. Mater. 2015, 27, 1316-1326, doi: 10.1002/adma.201404794.
3. Yi, F.; Lin, L.; Niu, S.; Yang, P.K.; Wang, Z.; Chen, J.; Zhou, Y.; Zi, Y.; Wang, J.; Liao, Q.; et al. Stretchable-Rubber-Based Triboelectric Nanogenerator and Its Application as Self-Powered Body Motion Sensors. Adv. Funct. Mater. 2015, 25, 3688-3696, doi: 10.1002/adfm.201500428.
4. Fu, S.; He, W.; Tang, Q.; Wang, Z.; Liu, W.; Li, Q.; Shan, C.; Long, L.; Hu, C.; Liu, H. An Ultrarobust and High-Performance Rotational Hydrodynamic Triboelectric Nanogenerator Enabled by Automatic Mode Switching and Charge Excitation. Adv. Mater. 2022, 34, 2105882, doi: 10.1002/adma.202105882.
5. Zhang, C.; Liu, Y.; Zhang, B.; Yang, O.; Yuan, W.; He, L.; Wei, X.; Wang, J.; Wang, Z.L. Harvesting Wind Energy by a Triboelectric Nanogenerator for an Intelligent High-Speed Train System. ACS Energy Lett. 2021, 1490-1499, doi: 10.1021/acsenergylett.1c00368.
6. Ma, G.; Gao, F.; Zhang, M.; Wang, Y.; Gu, C.; Meng, F.; She, J.; Song, Y.; He, X.; Wang, D.; et al. An Endurable Triboelectric Nanogenerator for Wind Energy Harvesting Based on Centrifugal Force Induced Automatic Switching between Sliding and Rolling Modes. ACS Sustain. Chem. Eng. 2024, 12, 12956-12965, doi: 10.1021/acssuschemeng.4c04516.
